# Extracellular Vesicles and Hepatocellular Carcinoma: Opportunities and Challenges

**DOI:** 10.3389/fonc.2022.884369

**Published:** 2022-05-25

**Authors:** Juan Wang, Xiaoya Wang, Xintong Zhang, Tingting Shao, Yanmei Luo, Wei Wang, Yunwei Han

**Affiliations:** ^1^ Department of Oncology, The Affiliated Hospital of Southwest Medical University, Luzhou, China; ^2^ Clinical Medicine, Southwest Medical University, Luzhou, China; ^3^ Department of Oncology, The Affiliated Hospital of Southwest Medical University, Nuclear Medicine and Molecular Imaging Key Laboratory of Sichuan Province, Academician (Expert) Workstation of Sichuan Province, Luzhou, China; ^4^ Nuclear Medicine and Molecular Imaging Key Laboratory of Sichuan Province, Luzhou, China; ^5^ School of Basic Medical Sciences, Shandong University, Jinan, China

**Keywords:** hepatocellular carcinoma, tumor microenvironment, hypoxia, vesicle drug delivery, extracellular vesicles

## Abstract

The incidence of hepatocellular carcinoma (HCC) is increasing worldwide. Extracellular vesicles (EVs) contain sufficient bioactive substances and are carriers of intercellular information exchange, as well as delivery vehicles for nucleic acids, proteins and drugs. Although EVs show great potential for the treatment of HCC and their role in HCC progression has been extensively studied, there are still many challenges such as time-consuming extraction, difficult storage, easy contamination, and low drug loading rate. We focus on the biogenesis, morphological characteristics, isolation and extraction of EVs and their significance in the progression of HCC, tumor invasion, immune escape and cancer therapy for a review. EVs may be effective biomarkers for molecular diagnosis of HCC and new targets for tumor-targeted therapy.

## 1 Introduction

China is the world’s top liver cancer country, and the 2020 Global Oncology Report showed that 906,000 patients of liver cancer occurred worldwide, of which 410,000 new cases occurred in China, accounting for >45% (1, 2). HCC is a common and fatal cancer, accounting for approximately 90% of all liver cancer cases (3). Although much progress has been made in diagnostic and treatment of HCC, such as liver excision, chemotherapy embolism and Sorafenib, it remains a health problem worldwide, with the incidence expected to exceed one million cases in a few years, due to its metastatic nature, high recurrence rate and low long-term survival (4, 5). EVs exist in tissues, various body fluids and supernatant, such as saliva (6), pleural effusion (7, 8), plasma (9, 10), urine (11), breast milk (12, 13), cerebrospinal fluid (14) and ascites (15, 16), which are greatly released by a variety of cells in a constitutive or inducible manner. EVs can regulate many biological processes, such as migration and extracellular matrix remodeling (17). Recently, some studies have shown that EVs play an important part in regulating cell signaling. Particularly, HCC cell-derived EVs may lead to local spread, distant metastasis and multifocal growth (18). HCC cell can secrete more EVs and promote tumor metastasis. After exposure to anti-tumor drugs, the release of EVs from hepatoma cell also increased, which activate natural killer cells and induce anti-tumor immunity. Besides, tumor cell-derived EVs can produce direct immune effects to stimulate target cells. It has been reported that EVs-mediated intercellular transfer may promote the invasion of HCC by affecting the tumor microenvironment (TME) (18, 19). EVs-mediated signaling in liver disease makes them a unique therapeutic tool that can provide targeted delivery of tissue siRNAs, miRNAs and circRNAs to affect gene expression (20). Notably, EVs are natural nanomaterials. Compared with drugs, modified EVs have many advantages, which significantly improve the specificity, efficacy, and safety of EVs-based cancer therapies and become ideal candidates for drug development and delivery (20). Nowadays, the use of biogenic EVs as drug delivery has become a research hotspot, and its complex phospholipid membrane structure may be conducive to immune escape, site-specific transmission, cell uptake and intracellular transport (21). In addition, some microRNAs in EVs have also been introduced as potential biomarkers, and their expression level is related to the invasiveness of HCC (22). It has been reported that EVs play a key role in biological functions, including intercellular transfer, angiogenesis, immune response, tumor growth and metastasis of HCC (23–25).

## 2 Introduction of EVs

### 2.1 Biogenesis and Morphological Characteristics of EVs

It is known that EVs can be a key role in human physiological and pathological diseases with various subtypes of cell-released membrane structures. EVs of particle diameters <200 nm are referred to as small EVs (sEVs) and medium-to-larger particles of diameters >200 nm are referred to as m/lEVs (26). Depending on the description of conditions or cell of origin, EVs can also be classified as apoptotic body, large oncosome, hypoxic EV, podocyte EV, etc, which are showed as follows (22, 26–28) (**Table 1**).

**Table 1:** The types of EVs.

Classification	Subtypes	Diameter	Source	Marker	Ref
Physical characteristics/Size	sEV	< 200nm	Originates from the inward outgrowth of multivesicular bodies (MVB), endosomal system	Transmembrane proteins CD9, CD63 and CD81; ALIX; TSG101	(5, 22, 29)
			Derived from hepatocytes, macrophages, NK cells, T cells, B cells		
	m/lEV	> 200nm	Plasma membrane outward budding production	Integrin; Selectin; CD40; Most membrane-associated proteins in source cells	(30, 31)
			Derived from almost all healthy living cells.		
Descriptions of conditions/Cell of origin	Apoptotic body	1-5μm	generated from cell fragments undergoing apoptosis	Phosphatidylserine; Genomic DNA; It is similar to the surface markers of its derived cells and rich in caspases-3 and caspases-7	(30, 32)
	Large oncosome	1-10μm	originates from the shedding of the membrane bubbles	CK18	(33–36)
			released by Invasive prostate cancer cells, urinary bladder, and glioblastoma		
	Hypoxic EV	–	Hypoxic cell	include mRNA and proteins (MMPs, IL-8, PDGFs, caveolin 1, and lysyl oxidase, etc)	(37, 38)
	Podocyte EV	–	from the tip of the microvilli of the podocytes	—	(39)

The sEVs (<200nm) originate from the inward outgrowth of endosomal membranes, are one of subpopulations of EVs (30), which can be produced from different cells such as hepatocytes (40), NK cells (41), T cells (42), and B cells (43), and surface markers of sEVs include CD9, CD63, CD81, and CD82 (44). sEVs are formed by the endonuclear body system and transmit information to the recipient cell through three main processes:First, the cytoplasmic membrane is initially invaginated by lipid raft-mediated endocytosis to form endocytic vesicles, which fuse with each other to form early endosomes (Endocytosis) (45);Second, early intranuclear bodies regenerate and invaginate, and intracellular material forms multiple intraluminal vesicles (ILVs), which are further transformed into late intranuclear bodies and multivesicular bodies (MVBs). This process also involves the inversion of cytoplasmic contents, transmembrane proteins, and peripheral proteins (Receptor-ligand Interaction) (46). Finally, MVBs fuse with the cytoplasmic membrane to form sEVs (Fusion With the Plasma Membrane) (5, 23, 47). In addition, MVBs have also been reported to fuse with lysosomes and promote the degradation of vesicle contents (27, 44, 48). The formation, release and sorting of sEVs are a series of regulated processes, which mainly require the endosomal sorting complex required for transport (ESCRT), members of the ESCRT family [apoptosis contiguous gene 2-interacting protein X (ALIX), also called PDCD6IP (49), tumor susceptibility gene 101 (TSG101)] (50, 51), four transmembrane proteins family (49, 52) and lipid raft-associated proteins (53, 54) and many substances are involved. As we all know, ESCRT is composed of ESCRT-0, ESCRT-I, ESCRT-II and ESCRT-III (55), and is associated with delivery of ubiquitinated proteins, degradation of lysosomes and recycling of proteins (20). Moreover, ESCRT plays an important part in luminal vesicle biogenesis and cargo aggregation (49). ESCRT-independent processes also seem to be involved in the formation and secretion of sEVs in an intertwined manner (56). Intracellular transport of sEVs involves many molecular switches, such as RAB GTpase proteins, membrane linked proteins, actin and microtubulin (23). Besides, Rab family proteins,including Rab7, Rab11, Rab27, and Rab35, also play a crucial role in the process of sEVs secretion (25). The secretion of sEVs also requires the involvement of the SNARE complex and the synaptic binding protein family (30). Furthermore, the involvement of sphingomyelinase in vesicle release was confirmed by the elevated ceramide levels in sEVs and less release of sEVs after sphingomyelinase inhibition (20, 56). Overall, sEVs regulate signaling pathways in receptor cells, coordinate TME and communication between different cells.

The m/lEVs (>200nm) are released by the plasma membrane to the outgoing buds, so the membrane composition of the m/lEVs is extremely close to the plasma membrane. The cell membrane surface is full of phosphatidylserine and most of the membrane-associated proteins, which can regulate the intercellular information exchange and affect the functions of target cells (30). The mechanism of m/lEVs formation is related to intracellular calcium signaling stimulation (23, 57), membrane bending proteins and the asymmetric distribution of phospholipids. The inward flow of calcium ions in the cytoplasm activates phospholipid crawling enzymes to disrupt phospholipid asymmetry, leading to redistribution of phospholipids in the cell membrane bilayer (58). The junctional protein ARRDC1 recruits ESCRT proteins and VPS4 (an ATPase) to the cell membrane (59); ESCRT-1 protein interacts directly with inhibitory proteins; pro-caspase3 stimulates Rho-related protein kinase 1 to promote apoptogenesis and induces myocardin contraction, contributing to the release of m/lEVs.

Apoptotic body (50-2000 nm), also known as apoptotic vesicles, are produced by debris cells that undergo apoptosis (60, 61). When cells undergo apoptosis, the cell membrane folds inward and wraps around the cytoplasm, organelles and nuclear fragments to form vesicles, which are the largest subpopulation of EVs. Apoptotic vesicles have surface markers and are enriched in caspases-3 and caspases-7, caspases-3 and Rho/Rock pathway taking part in membrane blistering (30, 32, 62). Moreover, apoptotic vesicles play a key role in attracting phagocytes, promoting the clearance of apoptotic cell debris, and regulating antigen presentation and immune cell responses (30). Apoptotic cells have been reported that can facilitate the encapsulation of chemotherapeutic drugs or nanoparticles into EVs (22). In addition, apoptotic vesicles from apoptotic cells can be preferentially taken up by macrophages and produce antitumor effects (22). Thus, apoptotic vesicles may also be an ideal delivery system, but the use of apoptotic vesicles as therapeutic nanovesicles (NVs) has been less studied, which may be related to their large cell size and uneven distribution.

Large oncosomes(1-10 μm) are released by cancer cells and may play a role in the tumor microenvironment. It has been shown that CK18 is a marker of large oncosomes and can be identified in circulation and tissues (63).

The mechanism of production of hypoxic EVs may depend on hypoxia-inducible factors and RAB22A, which in a hypoxic environment relies on the mediating action of the small GTPase RAB22A to dislodge hypoxic EVs from the cells (38). Hypoxic EVs are influenced by the environment and containing biomarkers such as mRNA and proteins, among which proteins include MMPs, IL-8, PDGFs, caveolin 1, and lysyl oxidase (37).

Podocyte EVs (100-200 nm) derived from the tip vesicles of podocyte microvilli (39). It can be expressed before other markers of nephropathy and therefore may serve as a new marker of glomerular and tubular injury.Medeiros et al. have shown that EVs can be produced by podocyte cells after exposure to high glucose and expressed before proteinuria (64). It remains to be proven about the biomarkers contained in EVs produced by podocytes.

### 2.2 Contents of EVs

**Figure 1:**
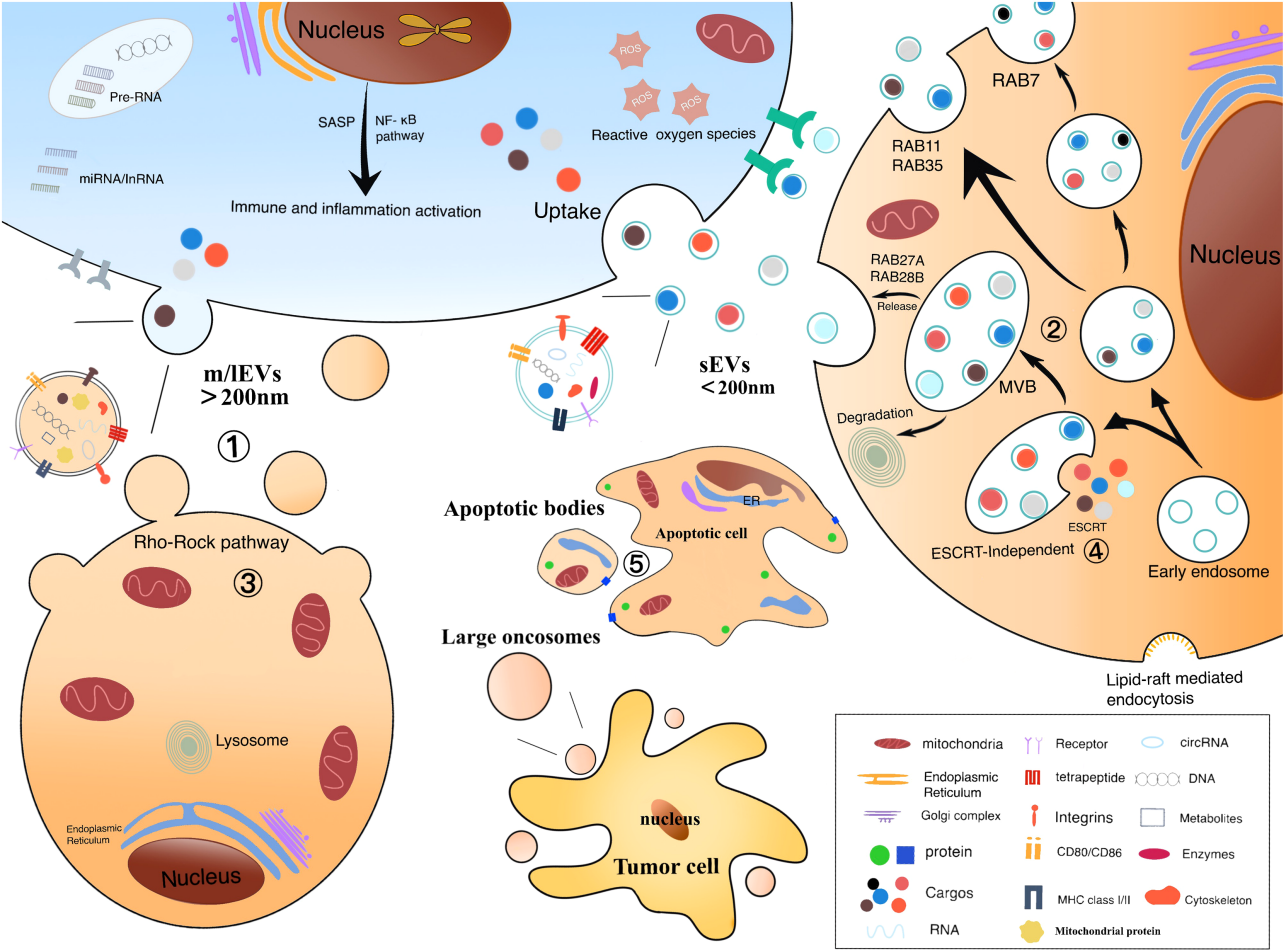
Biological origin of electric vehicles: ① m/lEV formation is the result of mass membrane foaming. Calcium relies on the cellular scale of protein hydrolysis degrading membrane binding, which can help cell membranes germinate and promote their secretion. ② Formation of sEV includes endocytosis, the formation of nucleosomes and MVBs, and the release of sEVs. The vesicles contained in MVBs fuse with the plasma membrane, causing their release. ③ Refactoring is related to the Rho/Rock pathway. ④ Composition of ESCRT is related to the biological occurrence of sEV and MVB. Rab protein facilitates the transport and docking of MVBs over the plasma membrane, leading to cytoplasmic vomiting and the release of sEVs. ⑤ Extensive membrane vesicles occur on the membrane of apoptotic cells to form apoptotic body.

EVs are usually secreted under physiological conditions and rich in nucleic acids, proteins, lipids, and metabolites (31) (**Figure 1**). In response to stimuli such as differentiation, neuronal signaling or immune response, the secretory content varies depending on the cells of EVs origin and their function. Surface proteins were abundant, with high enrichment of tetraspanins (CD9, CD63) and lysosome-associated membrane protein 2b (Lamp2b) (20). Besides, RNA is presented in EVs, including miRNA, long non-coding RNA (lncRNA), transfer RNA (tRNA), etc, which range from approximately 25 to 700 nucleotides in length and vary in content depending on the different origin of EVs (5). To be interest, EVs from tumor cells are particularly rich in RNA. According to the Vesiclepedia database, 213 unique proteins were identified in HCC cell-derived EVs. The sEVs proteins include cargo proteins and membrane proteins, the latter being associated with exocytosis of recipient cells and target organ selection (65, 66). The composition of cargo proteins in sEVs varies across tumor cells (5). Studies have found that the ultraconserved lncRNA (ucRNA) expression is dramatically altered within EVs as compared to donor cells. For example, HCC cell-derived EVs transfer ultraconserved lncRNA TUC339 enrichment to neighboring cells in the microenvironment, which is transcribed in host cells and promotes HCC proliferation and diffusion (66). In addition, Yang, B et al. suggested that EVs promote hepatocellular carcinoma metastasis because some substances in EVs are involved in epithelial mesenchymal transition (EMT) (40).

Moreover, many studies have reported that mitochondrial proteins are also cargoes of EVs (67–70). EVs can carry mitochondria, mitochondrial proteins, or mitochondrial DNA to travel between organelles (67, 71). Kiran Todka et al. found that mitochondrial proteins are selectively enriched in EVs and that delivery of mitochondrial proteins to EVs requires sorting nexin 9(SNX9)-dependent mitochondria-derived vesicles (MDVs). MDVs are responsible for carrying mitochondrial proteins between mitochondria and other organelles (72). Intercellular transfer of mitochondria (including mtDNA) results in altered mitochondrial function. If mitochondria are localized within the mitochondrial network of the recipient cell, it may elevate the intracellular ATP levels, further generate metabolic stress and ROS to regulate innate immunity, which may have a significant impact on the tumor microenvironment (73–75). For example, it has been found that mitochondrial DNA (12S rRNA (RNR1) G709A) play an important role in the development of HCC (76). However, whether the process of mitochondrial can influence the hepatocellular carcinoma progression associated with EVs needs to be further explored.

### 2.3 Specific Mechanisms of Uptake and Internalization Between EVs and the Target Cells

Since our current knowledge about the physiology, diversity, internalization, and cargo delivery of EVs is still somewhat limited, it remains impossible to derive a clear mechanism about how EVs interact with and modify receptor cells. However, determining the intracellular pathways and mechanisms of their cargo delivery could help us to utilize EVs as therapeutic agents appropriately (77).

The uptake pathways of EVs are known to be greatly diverse by cells and EVs type, which may be more dependent on the receptor cell type than EVs itself (22, 78, 79). EVs can translocate their contents to recipient cells by different mechanisms such as direct fusion, direct binding, endocytosis or phagocytosis (22). Although the mechanism of EVs uptake and cargo translocation into the cytoplasm of the receptor cell is still not fully defined, it mainly occurs in three steps: targeting the receptor cell, entering point into the receptor cell, and delivering the contents to the receptor cell. However, the end point of EVs internalization is still uncertain, and the function of EVs-mediated cargo transfer cannot being well defined (78).

The pathway of EVs internalization determines the functional response and efficiency of cargo delivery, while the internalization of EVs is mediated by a variety of mechanisms (80), including grid protein dependence and endocytosis of grid protein non-dependent pathways (78). In general, endocytosis is usually divided into two main subgroups: phagocytosis and cytokinesis. Phagocytosis is a type of endocytosis of relatively large (>1µm) particles and is usually restricted to specialized professional phagocytes. In contrast, all cells are capable of cytokinesis (81–83). Grid protein-mediated endocytosis is a recognized pathway for extracellular substance uptake (84). Meanwhile, studies have shown that EVs enter cells mainly through grid protein-independent endocytosis and macrocytosis (83). Non-dependent endocytosis of grid proteins, including the formation of inverted influxes of vesicle-coated cells on cell membranes (77, 84, 85). Alternatively, fusing with the exoplasmic membrane, EVs can enter cell directly, thereby release their contents into the cytoplasm (80).

## 3 Separation Methods of EVs

**Figure 2:**
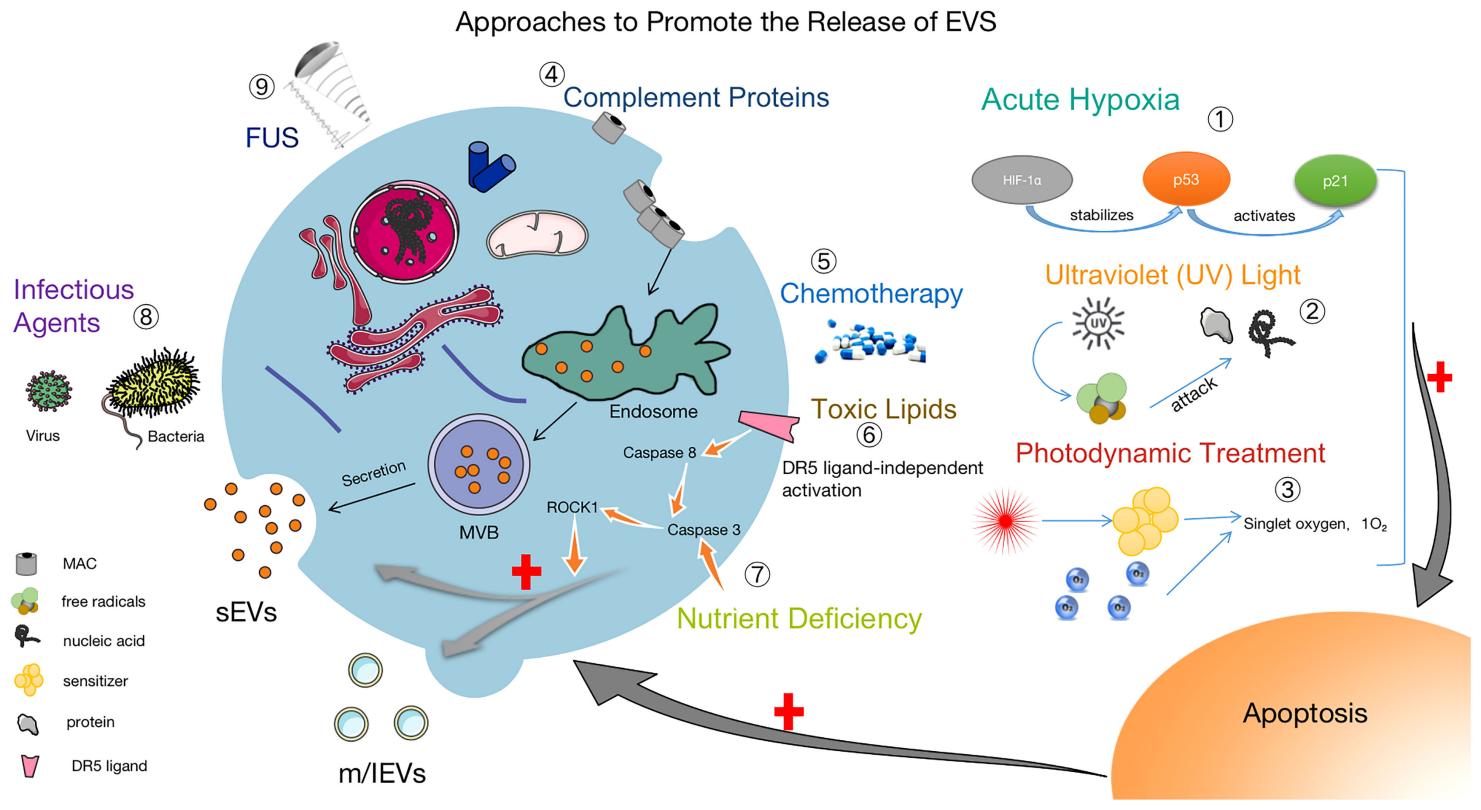
Methods to facilitate the release of EVs. ① Acute Hypoxia: Catabolism of HIF-1α is inhibited by acute hypoxia, which stabilizes the P53 gene and activates the P21 gene, leading to apoptosis and promoting the release of EVs (91–93). ② UV: After UV irradiation, a large number of free radicals are generated to attack nucleic acids and proteins, causing apoptosis and increasing the release of EVs (94). ③ Photodynamic Treatment: Laser irradiation at a specific wavelength excites the tissue-absorbing photosensitizer, and the excited state of the photosensitizer transmits energy to the surrounding oxygen, generating strongly reactive monomorphic oxygen, which may reacts oxidatively with the surrounding neighboring biomolecules, resulting in a cytotoxic effect that causes apoptosis and also promoting the release of EVs (95). ④ Complement Proteins: The membrane attack complex (MAC) is composed of complement proteins (C5b, C6, C7, C8 and C9). MAC is cleared from the cell surface by cytosolic or cytocytic action to help release EVs (96, 97). ⑤ Chemotherapy: The use of chemotherapeutic agents (e.g., doxorubicin, methotrexate, and cisplatin) causes cellular damage and EVs release (95, 98). ⑥ Toxic Lipids: Toxic lipids activates the DR5 pro-apoptotic signaling cascade, which in turn activates ROCK1 and promotes the release of EVs from hepatocytes (90). ⑦ Nutritional Deficiency: Activation of Caspase 3, ROCK1 signaling pathway and promotion the release of EVs (99). ⑧ Infection factors (100) and ⑨ focused ultrasound (101) can also promote the release of EVs.

The isolation and collection of EVs is a necessary condition for biomedical research and clinical transformation. Researchers have developed many methods to separate EVs, and it is particularly significant to use the proper isolation method under different conditions. For better clinical applications, improving existing technologies for the isolation and storage of EVs are facing great challenges (20). Efficient access to EVs is extremely important for research, and in addition to the use of suitable isolation techniques, promoting the production and release of EVs is also of great value. Upon increased release of EVs, cargo and surface marker proteins may cause altered biological functions (86, 87). Notably, EVs induced by tapping membrane complexes have been reported to play important physiological roles in enhancing immunity, promoting coagulation, wound healing and growth (88, 89). Hirsova, P found that toxic lipids induce the release of EVs from hepatocytes and can activate the pro-inflammatory response of macrophages, which also suggests that inhibiting the release of EVs could be a therapeutic strategy for patients with NASH (90). Based on the therapeutic potential of EVs, we believe that it is of great interest to select suitable methods to facilitate or inhibit the release of EVs depending on the purpose. Thus, some approaches to promote the release of EVs are summarized in **Figure 2**.

### 3.1 Traditional Methods

#### 3.1.1 Ultracentrifugation

Ultracentrifugation is considered as the “gold standard” for the separation of EVs (102). Due to the different particle size and density, its settling speed is also different, using gradually increasing centrifugal speed or low speed and high speed alternate centrifugation, can be separated in batches at different separation speeds and centrifugal time (30). Cellular impurities were removed with a low speed of 300 g, and high centrifugal force of 16,000 g can be used to separate apoptotic bodies, 20,000 g to separate m/lEVs, and 100,000 g to precipitate and concentrate sEVs (103, 104). This method is widely used, but the purity of sample is not satisfied for the supernatant will contain 40% EVs, which leads to protein contamination and lower yield. There is an overlap in the size of sEVs and m/lEVs, and slightly larger sEVs and smaller microvesicles are difficult to isolate (105). In addition, it generally requires multiple centrifugation processes to achieve better separation, but it is prone to vesicle destruction and also has many disadvantages such as the large size of the instrument, high cost, lengthy and laborious processing, and few samples (106).

#### 3.1.2 Gradient Ultracentrifugation

The requirements of gradient ultracentrifugation are more stringent, when there is a small difference in settling velocity between different particles, they are placed on the top of a medium with different density gradient. Under the action of a certain centrifugal force, the particles are separated by aggregating into the layer of the medium with a similar density to theirs, and the commonly used medium is sucrose (107). Sucrose gradient centrifugation can be used to isolate sEVs (108, 109). This method is popular because of good separation effect, high purity, no extrusion and deformation of the particles, and the ability to maintain the activity of the particles. However, it needs to prepare inert gradient media solution, be complicated to operate, not easy to master, time-consuming and labor-intensive (20-24 h), and high cost. What’s more, the density of EVs and high-density lipoprotein particles (HDL) is similar and they can be separated out together, so the samples are prone to contamination (110). Besides, the use of newer isotonic gradients contribute to better maintenance of the physical properties of the vesicles (111).

#### 3.1.3 Precipitation Method

The precipitation method mainly includes polymer precipitation and organic solvent precipitation. Commercial kits that rely on polymer co-precipitation have been reported being used for the isolation and purification of EVs, decreasing solubility and promoting precipitation. The precipitated EVs can be easily and reproducibly separated and avoid prolonged ultracentrifugation (112, 113). Unfortunately, the main problems with this method are that co-precipitation is susceptible to contamination by non-EVs substances and that mechanical forces or chemical additives can damage EVs (114). In addition, the method relies more on manual manipulation with low throughput and recovery, and purification of polymers from EVs may interfere with downstream analysis. Therefore, co-precipitation is not suitable for most research and clinical applications.

#### 3.1.4 Molecular Exclusion Chromatography

The principle of molecular exclusion chromatography is that different solute molecules, such as EVs and protein impurities, are separated from each other as they pass through porous packings due to differences in size resulting in different rates of passing through the pores (115, 116). This approach yields purified EVs from complex biological media (117–119), removed soluble plasma proteins and HDLs effectively, preserved the biological activity and integrity of EVs and also reduced aggregation (115). A variety of influencing factors such as media type, pore size, column size, and flow rate should be considered for EVs separation (20, 116). This method is efficient and inexpensive, and it is more suitable for small volumes of blood samples because of the upper sample volume limitation.

#### 3.1.5 Asymmetric Flow Field Flow Classification Method

Asymmetric flow field flow fractionation (AF4) is a technique in which a force field is applied to achieve the separation of EVs with different sizes and molecular weights (120). AF4 contains permeable plates and when a vertical force field is applied, the analytes in the sample will be moved to the boundary by the force and smaller particles will undergo Brownian motion to reach a new equilibrium position (121). The advantages of this method are rapid (<1 h), high resolution, gentle, label-free, and reproducible. It can be applied to a variety of eluates, contributing to the successful separation of different subpopulations of EVs.

### 3.2 New Methods

#### 3.2.1 Immunoaffinity Capture

Obviously, EVs are rich in proteins. Immunoaffinity capture is the specific binding of antibodies to the corresponding antigens on the surface of EVs such as adhesion proteins, tetra-transmembrane proteins and integrins, achieving the separation of EVs by immune reactions (122, 123). Magnetic beads provide a large surface area to capture EVs, targeting antigens on the surface of EVs to select specific subgroups, improving separation efficiency, specificity and purity, making it more suitable for marker detection of EVs and clinical diagnostic studies (124). However, the expensive antibody reagents, stringent reaction conditions, reduction of isolation yields, and the vulnerability of the biological activity of the EVs contents to PH and salt concentration have made it inappropriate to isolate large volume samples (125).

#### 3.2.2 Microfluidics

Based on different molecular size, microfluidics can isolate EVs from large cellular debris (126). Compared to conventional separation methods, with smaller sample volumes (50µL - 500µL), microfluidic techniques are faster (30 min-2 h), portable, cost effective and automated, resulting in high purity of EVs. However, some microfluidic technologies allow only small sample input, lack method validation and standardization, which may influence the application of downstream analysis.

#### 3.2.3 Contactless Classification

The use of acoustic waves for contactless separation of EVs has recently been proposed by some researchers. This separation method applies forces based on the size and density of vesicles (127). Particles in the acoustic region migrate toward the pressure nodes after the force is applied. Acoustic interaction forces are proportional to vesicle volume, with larger vesicles moving more rapidly. This method can separate EVs very quickly and without contact.

## 4 Quantification Methods of EVs

Currently, the quantification of EVs has been challengig. It is suggested that for conditioned medium, the number of cells at the time of initiation and collection should be clearly indicated. In addition, proper characterization of EVs at the time of separation helps to understand their properties. Several techniques for measuring the size of EVs are being investigated, including lateral-flow immunochromatographic assay (LFIA), nanoparticle tracking analysis (NTA), and nanopore tunable resistive pulse sensing techniques(TRPS), high resolution flow cytometry, multi-angle light scattering coupled to asymmetric flow field-flow fractionation (AF4), fluorescence correlation spectroscopy (FCS), enzyme linked immunosorbent assay (ELISA) and Raman spectroscopy, etc. Here, we talk about some advantages and disadvantages of some techniques. LFIA, with its high degree of flexibility, is a good tool for cost-effective field detection, but the assay lacks sensitivity (128). The AF4 system is highly repeatable (120), however, it requires skilled operators. NTA and TRPS can be used for particle size analysis of EVs, and their detection sensitivity is 70-90 nm and 70-100 nm, respectively. NTA technology allows one-time measurement and quantification of EVs, but the equipment is expensive and difficult to operate (129, 130). The ELISA technique is greatly flexible and can be modified appropriately for the analyte, but it is also time-consuming.

In addition, EVs are rich in proteins, lipids, nucleic acids and other biomolecules, and it can be quantified by quantifying these specific molecules. For example, total protein amounts were determined by using Bradford, micro-bicinchonic acid (BCA), fluorimetric assays, global protein stainon sodium dodecyl sulfate polyacrylamide gel electrophoresis (SDS-PAGE), etc. However, due to the possible presence of protein contaminants, the measurements are on the high side. The amount of total lipids can be measured by sulfofphosphovanilin assay (131) and total reflection fourier-transform infraredspectroscopy (132). RNA can be quantified by global RNA assays (133). In conclusion, the quantification of EVs is a critical topic that still lacks consensus and standardization both domestically and internationally, and we expect more studies to be reported in the future.

## 5 Interactions Between HCC and HCC Cell-Derived EVs

In the microenvironment where tumor cells and normal cells are located, HCC cell-derived EVs build a bridge to communicate with each other and promote HCC proliferation, invasion and distant metastasis, etc. EVs origined from HCC often regulate tumor progression through autocrine and/or paracrine cellular communication. HCC cell-derived EVs stimulate recipient cells to produce cytokines and promote the migration of HCC, such as matrix metalloproteinase 2 (MMP2) and matrix metalloproteinase 9 (MMP9) (134). Meanwhile, HCC is a typical hyperangiogenic tumor. HCC cells secrete EVs loaded with different miRNAs, LncRNAs, circRNAs that can activate signaling pathways in the recipient cells, thus causing the recipient cells to respond, promoting HCC migration or inhibiting HCC proliferation, which have an impact on tumor angiogenesis (47). For example, HCC cell-derived EVs carry oncogenic RNAs and proteins, which allows EVs to activate the PI3K/AKT and MAPK signaling pathways and promote distant tumor metastasis (46).

EVs secreted by HCC cells containing some specific miRNAs will play a specific role in HCC. For example, hypomethylation causes increased expression of mir -429 in HCC cells, and these large EVs mediated by mir -429 are shed and bind to Rb-binding protein 4 (RBBP4) in surrounding target cells, promoting the transcriptional activity of E2F1 and ultimately upregulating the expression of POU class 5 homeobox 1 (POU5F1) in target cells, thereby promoting HCC development (46). Meanwhile, EVs-loaded miR-221 binds to the 3’-UTR target site of the p27/Kip1 oncogene and promotes HCC proliferation and migration (135). EVs containing protein CD147 released by HCC cells activate the NF-κB pathway of surrounding fibroblasts, induce MMP-9 expression, and stimulate the ERK1/2 and p38 MAPK pathways, leading to extracellular matrix degradation and tumor invasion (136, 137). In addition, EVs containing miR-25 released from HCC cells inhibited p53 expression in surrounding HCC cells, thereby restoring FOXM1 (a key regulator of cell cycle progression) expression, activating the HGF/Ras pathway, reversing the expression of sorafenib-induced apoptotic markers BCL2 and BAX, making HCC cells resistant to sorafenib (138). miR-34a is reduced in the large EVs released by CHB or HCC cells, resulting in increased levels of mRNA and protein in c-Mets in surrounding cells, promoting phosphorylation of c-Met-induced extracellular signal- regulated kinases 1 and 2 (ERK1/2), thereby facilitating CHB conversion to HCC (139, 140). Intracellular TLR4 signaling in HCC cells is transduced to the actin cytoskeleton *via* the MyD88 pathway, leading to the release of large EVs. Peripheral tumor macrophages take up large EVs containing microRNA let-7b, which attenuates tumor inflammation by targeting the pro-inflammatory cytokine IL-6 (141). Upregulation of ANXA2 expression in HCC cells promotes the shedding of CD147-containing large EVs and the production of MMP-2 in surrounding fibroblasts, thereby promoting HCC development (142). Thus, HCC cell-derived EVs can also act as a bridge between surrounding tumor cells or other cells, and their loaded cargo can have an impact on HCC progression when taken up by target cells.

### 5.1 HCC Cell-Derived EVs Promote HCC Migration by Directly Activating or Inhibiting Signaling Pathways

**Figure 3:**
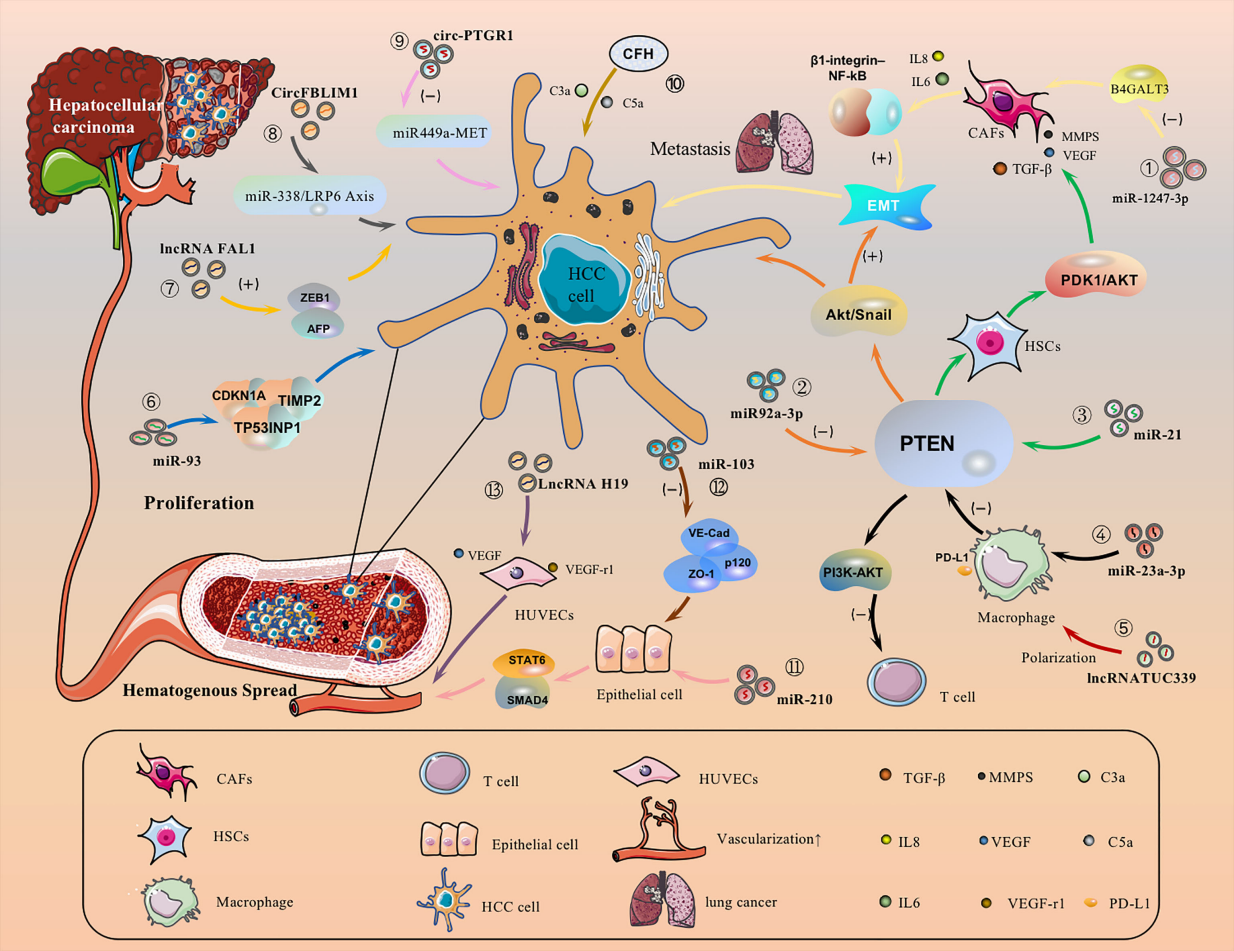
HCC cell-derived EVs carry cargo and regulate different receptor cells.

HCC cell-derived EVs-loaded cargoes can promote cancer cell migration by directly activating or inhibiting signaling pathways. For example, EVs-miR-1247-3p secreted by HCC cells directly transferred to lung pre-metastasisniche fibroblasts, decreased the expression of β-1,4-galactosyltransferases III (B4GALT3, a protein mediating glycosylation), thereby converting them into CAFs, and then activated the β1-integrin-NF-κB signaling pathway to promote EMT, thereby promoting the metastasis of hepatocellular carcinoma to the lung, and IL-6 and IL-8 secreted by CAFs to promote the development of HCC (**Figure 3**.①) (143). Meanwhile, EVs-miR92a-3p can promote HCC metastasis and EMT by inhibiting PTEN activation of the Akt/Snail signaling pathway (**Figure 3**.②) (40). Besides, HCC cells can also secrete EVs-miRNA-21 that directly targets PTEN and activates the PDK1/AKT signaling pathway. Moreover, it transforms hepatic stellate cells (HSC) into activated cancer-associated fibroblasts(CAF), which can further promote HCC growth by secreting vascular growth factors(VEGF, MMP2, MMP9 and TGF-β) (**Figure 3**.③) (144). Under endoplasmic reticulum stress, HCC cells inhibit PTEN and activate the PI3K-AKT pathway by delivering EVs-miR-23a-3p to macrophages, increasing macrophage PD-L1 expression and inhibiting T-cell function, promoting immune escape (**Figure 3**.④) (145). In addition, EVs-lncRNA TUC339 can be taken up by THP-1 cells, resulting in reduced production of pro-inflammatory cytokines, reduced expression of costimulatory molecules, impaired phagocytosis, and promotion of macrophage M (IL-4) polarization (**Figure 3**.⑤) (146). EVs-miR-93 promotes HCC tumorigenesis by affecting CDKN1A, TP53INP1, and TIMP2, and sEVs-miR-93 overexpression predicts poor prognosis (**Figure 3**.⑥) (147). It has been reported that lncRNA FAL1 are taken up by surrounding HCC cells and promote HCC cell proliferation and migration by competitively binding miR-1236 in recipient cells, which in turn upregulates the expression of their target genes AFP and ZEB1 (**Figure 3**.⑦) (148). sEVs-CircFBLIM1 can promote HCC progression through the miR-338/LRP6 axis (**Figure 3**.⑧) (149). The sEVs-circ-PTGR1 downregulates miR449a-MET expression, disrupts tumor microenvironment homeostasis, and promotes HCC migration and invasion (**Figure 3**.⑨) (150). EVs complement factor H (CFH) elevates C3a and C5a levels, exacerbating inflammatory responses and tumor growth (**Figure 3**.⑩) (151).

### 5.2 The Role of HCC Cell-Derived EVs on Angiogenesis

HCC is typically a highly angiogenic tumor and therefore angiogenesis is closely related to the prognosis. We have known that EVs-loaded cargo is able to promote angiogenesis and increase vascular permeability. Altered vascular permeability implies altered endothelial continuity, allowing cancer cells to infiltrate and attach to the microvascular endothelial lining and form tumor metastases. For example, Lin, XJ et al. found that delivery of EVs-miR-210 to endothelial cells to target SMAD4 and STAT6 for pro-angiogenesis (**Figure 3**.⑪) (152). Besides, EVs-miR-103 inhibits the expression of VE-Cad, p120 and ZO-1 and reduces endothelial integrity to promote tumor invasion (**Figure 3**. ⑫) (153). EVs-LncRNA H19 induces the production of the pro-angiogenic cytokine (VEGF) and its receptor VEGF-r1 in HUVECs and stimulates angiogenesis (**Figure 3**. ⑬) (154). Interestingly, Y Zhou et al. found that ovarian cancer-derived EVs carry NID1 through ERK/MAPK to promote EMT, accelerate angiogenesis, and promote tumor invasion (155), but the role of NID1 in HCC is still unclear (156). In addition, HCC cell-derived EVs can promote angiogenesis in HUVECs, and the amount of HepG2-derived EVs determines the amount of angiogenesis, lumen formation. The sEVs may influence human umbilical vein lumen formation *via* the VEGF receptor and the angiogenesis-associated heat shock protein HSP70 (157).

HCC cells-derived EVs carrying proteins were found to inhibit angiogenesis by reducing VEGF through activation of AMPK signaling iynamic network microenvironment consisting of hepatocytes and their surroundings, suchn HCC (158). At the genetic level, CLEC3B-related genes are closely associated with angiogenic genes. In experiments, cells with high levels of CLEC3B formed fewer vessels than those with low levels. Likewise, in animal studies, immunohistochemical detection of tumor tissue from *in situ* tumor-implanted mice showed a significant reduction in CD31-positive and CD34-positive endothelium (EC) in CLEC3B high-isogenic grafts. Thus, high levels of CLEC3B EVs significantly reduce the expression of endothelial growth factor (EGF) in HCC, thereby reducing angiogenesis.

### 5.3 Inhibition of HCC Growth by EVs-Loaded Cargo of Different Cellular Origin

When certain signaling pathways are blocked by EVs-loaded cargo, the growth and distant metastasis of HCC may also be inhibited. For example, when Vps4A is overexpressed in HCC cell-derived EVs, it inhibits the PI3K-Akt pathway and thereby inhibits the metastasis of HCC (159). When normal cells secrete sEVs containing SENP3-EIF4A1, SENP3-EIF4A1 inhibits HCC cell proliferation by suppressing miR-9-5p in HCC cells and activating the expression of ZFP36 (160). In contrast, EVs-circ-0051443 promotes HCC cell apoptosis and inhibits tumor growth by competing with miR-331-3p in HCC cells and upregulating BAK1 expression (161). Interestingly, Huang, X et al. proposed that IncRNA 85 regulates the invasion of cancer cell by targeting miR-324-5p and through ceRNA mechanisms, and more importantly, miR-324-5p overexpressed can reducing migration by regulating the expression of MMPs, ETS1 and SP1 genes in HCC (162, 163). When tumor-associated fibroblasts (CAFs) secrete EVs containing miR-320a, miR-320a inhibits HCC growth by suppressing the PBX3/ERK1/2/CDK2 pathway in HCC cells (164). For example, EVs enriched in LncRNA H19 were secreted by CD90+ cancer cells to promote angiogenesis, inducing the production and secretion of the pro-angiogenic cytokine VEGF and its receptor in HUVECs (154). What’s more, it has been shown that co-culture of Huh7 cells with HepG2 cells, where Huh7 secretes EVs containing miR-122, has an inhibitory effect on tumor growth, when co-cultured HepG2 cells attenuate this inhibitory effect by secreting IGF1 (165).

Alteration of original physiological functions between HCC cells through the delivery of cargo molecules in EVs. Some goods are markers to diagnose HCC from other liver diseases; Some can determine the effectiveness of HCC treatment and predict the recurrence rate of HCC; Some can be used as vehicles for delivering drugs for the treatment of HCC. In conclusion, EVs loaded with cargo play different roles in the migration of HCC, regulating the talks between HCC and cells (**Table 2**).

**Table 2:** The cargos and functions of EVs related with HCC.

Name of the Cargo in EVs	Cargo Type	Mechanism of the Cargo	Function of the Cargo	Vivo or vitro	Cell lines	Refs
miR-429	miRNA	Targeting the RBBP4/E2F1/OCT4 axis in recipient cells, promote liver T-ICs properties	Facilitate HCC	Vitro	T-ICs	(46)
miR-142-3p	miRNA	Down-regulation of RAC1	Suppressed migration of HCC	Vivo	Hepa1-6	(166)
miR-221	miRNA	Binding to the target sites in the 3’-UTR of p27/Kip1 tumor suppressor gene	Promote proliferation of HCC	Vitro	SMMC-7721	(135)
miR-25	miRNA	Attenuating p53 and enhancing FOXM1 expression	Mediate sorafenib resistance in HCC	Vitro	HepG2	(138)
miRNA let7b	miRNA	Targeting proinflammatory cytokine IL-6	Attenuates tumor inflammation	Vivo,Vitro	H22	(136, 141)
miR-34a	miRNA	miR-34a was down-expressed in HCC, promoted the translation of antiapoptotic factors	Promote the conversion of CHB to HCC	Vitro	–	(139)
CD147	protein	Induce upregulation of MMPs in fibroblasts, leading to extracellular matrix degradation	Promote tumoral invasion	Vitro	–	(136)
miR-1247-3p	miRNA	Targets B4GALT3, activate β1-integrin–NF-κB signaling, activated CAFs secrete pro-inflammatory cytokines	Promote lung migration of liver cancer	Vivo,Vitro	CSQT-2	(143)
miR-103	miRNA	Inhibiting the expression of VE-Cad, p120 and ZO-1, attenuated the endothelial junction integrity	Promote vascular permeability and metastasis	Vivo	MHCC97H	(153, 167)
miR-638	miRNA	Attenuate endothelial junction integrity	Promote vascular permeability and metastasis	Vivo	HuH-7M	(168)
miR-93	miRNA	Directly inhibiting the expression of TIMP2/TP53INP1/CDKN1A	Promote proliferation and metastasis of HCC	Vitro	SKHEP1	(147)
miR-23a-3p	miRNA	Promotes PD-L1 expression in macrophages and inhibits T-cell function through miR-23a–PTEN–AKT signaling pathway	Promote proliferation and metastasis of HCC	Vivo,Vitro	HepG2	(145)
lncRNAFAL1	lncRNA	Competitively binding to miR-1236, indirectly up-regulated the expression of AFP and ZEB1	Promote proliferation of HCC	Vitro	Huh7	(5, 148)
IncRNA 85	lncRNA	Targeted miR-324-5p and regulated its expression through a ceRNA mechanism	Promote proliferation and metastasis of HCC	Vitro	HepG2	(163)
lncRNATUC339	lncRNA	Excess lncTUC339 expression in macrophages promoted M(IL-4) polarization	Suppress the immune response to tumor cells	Vitro	HL-7702	(147)
circUHRF1	circRNA	Upregulate TIM-3 expression and suppress the production of IFN-γ and TNF-α	Inhibit NK cell function	Vivo	SMMC-7721	(169)
Vps4A	protein	PI3K/Akt pathway was inactivated by Vps4A-overexpression	Inhibit the growth and metastasis of HCC	Vivo	Hep3B	(159)
CFH	protein	Increase the production of C3a and C5a	Promote proliferation and metastasis of HCC	Vivo,Vitro	Huh7	(151)

## 6 Regulation of HCC by Different Cell-Derived EVs in the Microenvironment

There is growing evidence that the dynamic network microenvironment consisting ofhepatocytes and their surroundings, such as cancer cells, immune cells, cytokines andextracellular matrix is also a key factor in tumor metastasis. Liver is rich in immune cells, which can greatly produce EVs, and has a unique immune-tolerant microenvironment, which is a huge challenge for HCC immunotherapy (170). Among them, various immunosuppressive cell subsets and signaling pathway-mediated pre-tumor immune responses play a key role in “tumor immune escape”. EVs are not restricted by space and material and can interact with cancer cells anywhere in the body. EVs produced by cancer cells can also interact with nearby immune cells (171, 172). The interaction between tumor and the immune system determines the progression of the tumor at the early stage. In conclusion, HCC occurs not only because hepatocytes contain sufficient genetic mutations, but multiple interrelated factors in the hepatic microenvironment influence the progression of HCC, and the mechanistic features of these new factors have prompted the search for new therapeutic approaches to treat not only the tumor itself but also the hepatic microenvironment to prevent recurrence and treatment resistance, some of which have yet to be fully elucidated.

### 6.1 Mesenchymal Stem Cells-Derived EVs

**Figure 4:**
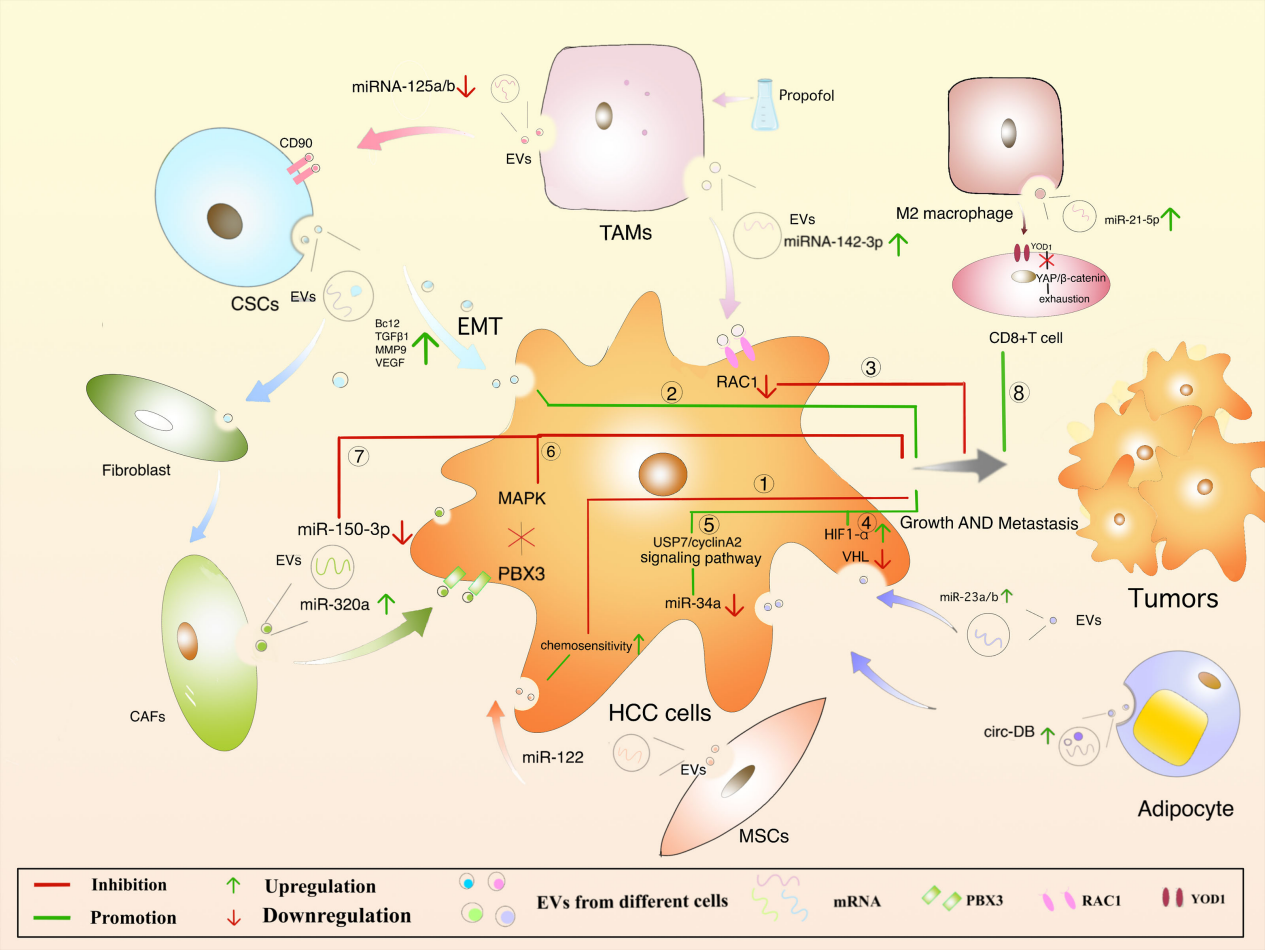
Regulation of HCC by different cell-derived EVs.

MSCs are present in bone marrow, umbilical cord blood and adipose tissue and are adult stem cells with multidirectional differentiation potential (173). MSCs attenuate fibrosis by upregulating hepatocyte growth factor (HGF) (174, 175), insulin growth factor (176), and MSCs-derived EVs improve hepatocyte regeneration and modulate immune activity, demonstrating therapeutic benefits in various liver diseases (173). Meanwhile, the role of MSCs-derived EVs cannot be ignored. Experiments have shown that ADMSC (adipose-derived mesenchymal stem cells)-derived EVs promote anti-tumor responses of NKT cells, leading to early ADC increase and low-grade tumor differentiation (177). In addition, an anti-tumorigenic effect of MSC-EVs was also observed in a CCl4-induced mouse liver tumor model. After treatment with EVs, the growth of liver tumor was significantly inhibited by inhibiting oxidative stress (178). Bruno, S et al. have demonstrated that EVs in human BM-MSCs can induce HepG2 cell cycle blockers and apoptosis necrosis *in vitro*, which inhibit tumor growth in the body. However, EVs secreted by fibroblasts formed by differentiation of human derived MSCs lack antitumor effects (179). In addition, miR-122 delivered *via* AMSC-derived EVs may provide new therapeutic options for HCC (**Figure 4**. ①) (180). It remains unclear that whether MSCs-derived EVs can inhibit HCC progression by carrying cargo, and it provides a new direction for the possibility of using MSCs-derived EVs as carriers to exert anti-tumor effects.

### 6.2 Cancer Stem Cells-Derived EVs

Cancer Stem Cells (CSCs), with proliferative and differentiation potential, is more easily contributing to tumor recurrence (181–184). It is reported that EVs derived from CSCs can induce tumor growth, metastasis, participating in angiogenesis and maintaining the stem cell phenotypes (185–188). EVs released from CSCs containing multiple cargoes, including proteins and multiple RNA (189). EVs can make the microenvironment to change in the direction of promoting tumor occurrence and metastasis. For example, Domenis, R et al. found that CSC- derived EVs inhibits T cells through monocyte-specific secretion of IL-10 (190). In addition, fibroblasts can be converted into cancer-associated fibroblasts (CAF) through the uptake of CSC-derived EVs, promoting tumor progression and metastasis (191). It was also found that CSCs-like CD90+ hepatocytes regulate the endothelial phenotype by releasing EVs containing H19 lncRNA, significantly increase VEGF expression, and promote intercellular adhesion, induce angiogenesis, and affect the tumor microenvironment (154). What’s more, Alzahrani FA et al. showed that hepatic CSCs-derived EVs were able to increase the expression of Bcl2, TGFβ1, NFκB, MMP9, VEGF, 13K, ERK and decrease the levels of Bax, p53, TIMP1 mRNA in the liver of mice, suggesting that CSCs-derived EVs promote hepatocellular carcinoma cell invasion while upregulating TGFβ1-induced EMT (**Figure 4**. ②) (192). However, it is of interest that CSCs-derived EVs and MSCs derived EVs had opposite effects on HCC growth and progression *in vivo*, and neither involved promotion or inhibition of HCC-induced oxidative stress or antioxidant activity. As can be seen, these studies have showed new insights into the treatment of HCC, and more research is needed to clarify the mechanisms involved.

### 6.3 Macrophages-Derived EVs

Depending on the state and functional status of macrophages after activation, they can be divided into M1 and M2 macrophages, with M1 macrophages playing a tumoricidal role and M2 macrophages promoting tumorigenesis (193). M1 macrophages are involved in the polarization of Th1 and high expression of IL-6, IL-12, TNF-α, iNOS, ROS to promote the occurrence of inflammation (194). EVs from M1 macrophages induce stronger antigen-specific cytotoxic T-cell responses in lymph nodes, enhance immune responses to cancer vaccines, and are used as effective vaccine adjuvants (195). In the TME, tumor-associated macrophage (TAM)-derived EVs significantly downregulate miRNA-125a and miRNA-125b (miRNA-125a/b targets CD90, a stem cell marker for HCC) and promote the progression of HCC (196). The macrophages were treated with propofol to help secrete more EVs with miRNA-142-3p, which can be absorbed by HCC cells, and furtherly, RAC1 inhibited the migration and tumor growth in mice (**Figure 4**. ③) (166). M2 macrophages are involved in Th2 polarization and highly express IL-4, IL-10, TGF-β, CD206, CD163, CCL22, etc., while reduce the expressing of IL-12,downregulate the immune response and promote tumor progression (197). In an experiment by Jian Pu et al. in which EVs were injected into a mouse model of liver cancer, M2 macrophage-derived EVs were found to promote CD8+ T cell failure *via* the miR-21-5p/YOD1/YAP/β-catenin axis (**Figure 4**. ⑧) (198). Thus, it seems that M2 macrophages are closely associated with the malignant development of HCC (199).

### 6.4 Adipocytes-Derived EVs

Adipocytes mainly play a role in providing metabolic substrates for tumor cells. There is evidence that adipose-derived EVs can promote tumor growth in HCC by downregulating VHL, delivery of miR-23a/b. Studies *in vivo* have shown that increasing levels of EVs-miR-23a/b, VEGF, GLUT1 and HIF1α accelerated tumor growth and rate in high fat diet mice (**Figure 4**. ④) (200). Visceral adipocyte exocytosis induces dysregulation of the TGF-b pathway in HepG2 cells in high body fat individuals, but not in low body fat individuals (201). Zhang, H et al. suggested that EVs-circ-DB was upregulated in HCC patients with high body fat and its positively correlated USP7 was also increased (202). Mature adipocyte-derived EVs and HCC cellular effects lead to a decrease in miRNA-34a (tumor suppressor), while an increase in the USP7/Cyclin A2 signaling pathway (pro-cancer), a promotion of HCC cell growth, and a reduction in DNA damage (**Figure 4**. ⑤). Nevertheless, once circ-DB is knocked out, these effects will disappear. Furthermore, adiponectin is an abnormally abundant adipocytokine that regulates sEVs biogenesis by binding to T-cadherin and reduces cytosolic ceramide levels by releasing EVs (203, 204). sEVs are formed through the non-dependent mechanism of ESCRT, a process in which ceramide is essential and accordingly lipocalin is crucial in regulating their exocytosis. sEVs as a biological delivery vehicle for cancer treatment has been a hot research topic recently, but the role of adipocyte-derived EVs in HCC still requires further investigation.

### 6.5 Fibroblasts-Derived EVs

The connective tissue is rich in fibroblasts. Understanding the regulation of CAF in HCC is critical. CAFs-derived EVs are low in miR-320a, which binds to its direct downstream target PBX3 and inhibits HCC by suppressing MAPK pathway activation (**Figure 4**. ⑥) (164). The expression of CAFs-derived EVs-MiR-150-3p is reduced, which can inhibit the migration and invasion of hepatocellular carcinoma cells (**Figure 4**.⑦) (205), suggesting it may be a new therapeutic option. Meanwhile, studies have reported that miR-195 in HCC has been downgraded to VEGF, CDC42, CDK1, CDK4, CDK6, and CDC25 (206, 207). As described, understanding the mechanism of fibroblasts-derived EVs on HCC can help design new therapeutic approaches.

## 7 Hypoxia-Induced Microenvironment Affects the Regulation of HCC by EVs

**Figure 5:**
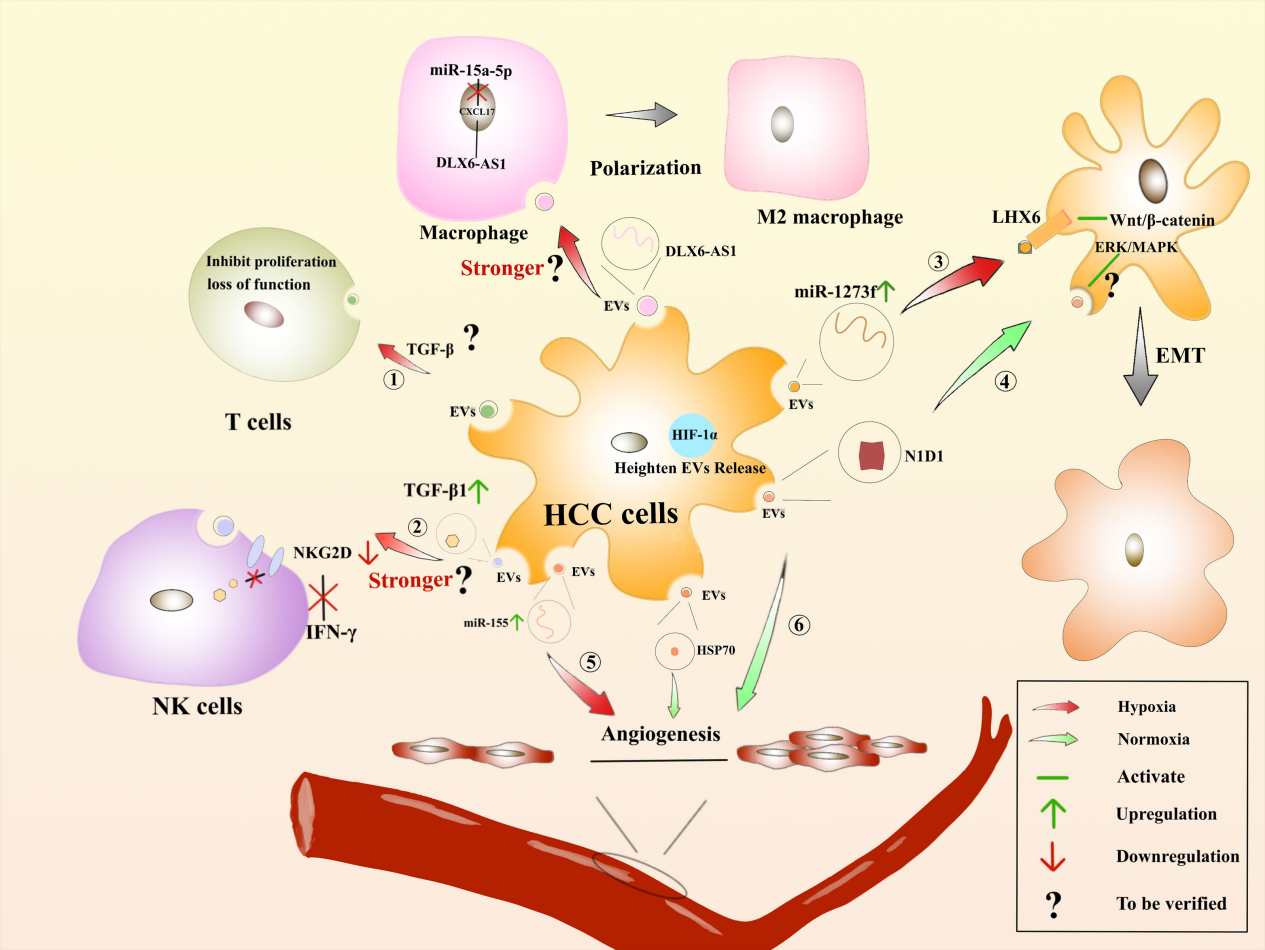
Hypoxia-induced microenvironment affects the regulation of HCC by EVs : The role of EVs derived from HCC on immune cells in the hypoxic environment. ① Suppressing the proliferation of T cells or rendering them incompetent. ② Whether the inhibitory effect on IFN-β production by NK cells and the process of inducing macrophage polarization are enhanced remains to be verified. The role of HCC-derived EVs facilitates EMT. ③ In the hypoxic environments, miR-1273f is upregulated in HCC-derived EVs, acting on LHX6 to activate Wnt/β-catenin to promote EMT. ④ In the normoxic environment, HCC-derived EVs contain N1D1, which may activate the ERK/MAPK pathway in recipient HCC cells to promote EMT. Regulation of angiogenesis by HCC-derived EVs. ⑤ In the hypoxic environments, miR-155 is upregulated in HCC-derived EVs and promotes angiogenesis. ⑥ In the normoxic environment, HCC-derived EVs are enriched in N1D1 and HSP1, which promote angiogenesis.

Many solid tumors live in the hypoxic microenvironment. Hypoxia promotes the production and release of EVs from cancer cells. Studies have showed that the number of sEVs in breast cancer cells and oral squamous carcinoma cells was significantly increased under hypoxic conditions (208). Hypoxia-inducible factor-α1 is a regulator of cells under hypoxic conditions and can facilitate the release of EVs (209). The proteins and nucleic acids of sEVs are also altered in the hypoxic environment (210). Under hypoxic conditions, miR-1273f carried by sEVs could accelerate the progression of HCC, targeting LHX6, which further inhibits HCC tumorigenesis or malignant transformation by targeting the Wnt/β-catenin signaling pathway (211). Hypoxia-generated sEVs can inhibit the expression of E-cadherin, thereby promoting EMT (212). EVs derived from HCC cells could affect angiogenic endothelial cells under the hypoxic conditions through upregulation of miR-155, thereby affecting tumor angiogenesis (213). Furthermore, EVs released from epithelial ovarian cancer (EOC) cells can express more miR-21-3p, miR-125b-5p and miR-181d-5p under the hypoxic conditions, thus facilitating M2 macrophage polarization (214). Additionally, DLX6-AS1 carried by HCC is in competition with miR-155 to regulate CXCL17. M2 macrophage polarization is induced, and migration, invasion, and EMT of HCC will be accelerated (215). Unfortunately, the authors did not investigate whether hypoxia accelerates this process. Rong, L et al. saying that hypoxia enhanced the secretion of sEVs in breast cancer cells, thereby inhibiting the proliferation of T cells (216). Moreover, hypoxia induced a significant increase in TGF-β1 content in cancer cell-derived EVs, decreased the expression of the activation receptor NKG2D, and inhibited the cytotoxicity of NK cells and also reduced the production of IFN-γ (217). Therefore, the tumor hypoxic microenvironment is closely related to tumor development, treatment and prognosis, which has become a research hotspot to find new treatments for HCC (**Figure 5**).

## 8 Biomarkers

EVs are providing important links for intercellular information transfer (218), and specific proteins and nucleic acids in EVs are important biomarkers for clinical diagnosis of various liver diseases.At present, the clinical assessment of liver damage is mainly based on liver enzyme profiles, such as aspartate aminotransferase (AST), alanine aminotransferase (ALT) (219–221). However, these enzyme markers lack specificity for liver diseases. Traditional tumor markers such as AFP, AFP-L3 are susceptible to other liver diseases and cannot analyze HCC for etiology, which has certain limitations. Therefore, to find new specific markers for patients with liver disease is significant. Much research mentioned that the proteins and nucleic acids carried by EVs can serve as markers to predict the prognosis of patients with liver disease (222–224).

### 8.1 EVs-Associated Nucleic Acids as Biomarkers for HCC Diagnosis

#### 8.1.1 miRNAs

miRNAs in serum EVs hold great potential as novel diagnostic biomarkers, and some of which have been reported worldwide (**Table 3**). Elevated levels of miRNA-21 and lncRNA-ATB expression were found to have higher specificity and sensitivity for HCC (232, 239). Patients with postoperative recurrence of HCC have significantly reduced the expression of miRNA-718, which was associated with the highly aggressive nature of HCC (233). Interestingly, Wang, Y et al. proposed that EVs-miR-122, EVs-miR-148a and EVs-miR-1246 in HCC patients serum were apparently higher than those in the LC and the NC group, and that these miRNAs combined with AFP could effectively reduce the rate of misdiagnosis (227). However, for HCC patients with low AFP expression, whether or not with hepatitis virus infection, sEVs’ miRNAs are more indicative of being markers of HCC when they are expressed as miR-10b-5p+ miR-221-3p+ miR-223-3p and miR-10b-5p+ miR-221-3p+ miR-223-3p+ miR-21-5p (234). Tian X et al. indicated that an acidic environment triggers HIF-1α and HIF-2α activation and facilitates the expression of EVs-miR-21 and EVs-miR-10b, significantly promoting the progression of HCC both *in vivo* and vitro (235, 249). We also find that several miRNAs are studied at high frequency, such as miR-21 and miR-122, and the results may differ in different study contexts. Besides, we read that some serum miRNAs are biomarkers of HCC (250–256), but it is not explicitly stated that these miRNAs are associated with EVs, and their roles in the progression and recurrence of HCC need to be further explored.

**Table 3:** EVs as biomarkers for the diagnosis of HCC.

Classification		Biomarkers	Expression	Species	Type of biological fluid	AUROC	Clinical significance	Refs
m/lEvs		AnnexinV+EpCAM+ASPGR1+CD133+taMPs	↑	Human	serum	0.7439	Diagnosis of HCC/CCA from LC	(225)
		EpCAM+AnnexinV +ASGPR1+taMPs	↑	Human	serum	0.7322	Diagnosis of HCC/CCA from LC	(225)
		Total m/lEVs of peripheral blood	↑	Human	serum	0.83	Diagnosis of E-HCC from LC (TNM stage I)	(226)
sEVs	microRNA	miR-148a	↑	Human	serum	0.871	Diagnosis of HCC from NC Diagnosis of E-HCC from LC	(227)
						0.860		
		miR-122	↑	Human	serum	0.990	Diagnosis of HCC from NC Diagnosis of E-HCC from LC	(227)
						0.795		
		miR-1246	↑	Human	serum	0.825	Diagnosis of HCC from NC Diagnosis of E-HCC from LC	(227)
						0.761		
		miR-638	↑	Human	serum	——	Associated with tumor recurrence, As a prognostic marker	(228)
		miR-125b	↑	Human	serum	0.739	Prediction of recurrence and survival	(229)
		miR-93	↑	Human	serum	0.825	The prognosis and diagnosis of HCC	(147)
		miR-665	↑	Human,	serum	——	Diagnosis and prognosis of HCC	(230)
				Mice				
		miR-92b	↑	Human,	serum	0.702	Prediction of E-HCC relapse after LDLT	(231)
				Rats				
		miR-21	↑	Human	serum	——	Detection of E-HCC, Prognostic marker	(232)
		miR-718	↑	Human	serum	——	Prediction of HCC relapse after LDLT	(233)
		miR-21-5p	↑	Human	serum	0.71	Diagnosis of HCC from LC	(234)
		miR-21, miR-10b	↑	Human,	serum	——	Prognostic markers of E-HCC	(235)
				Mice				
		miR-18a, miR221, miR-222, miR224	↑	Human	serum	——	Diagnosis of HCC from LC/CHB	(223)
		miR-101, miR106b, miR-122, miR-195	↑	Human	serum	——	Diagnosis of HCC from CHB	(223)
		miR-122, miR148a, miR-1246	↑	Human	serum	——	Diagnosis of HCC from LC	(227)
		miRNA-519d, miR-595, miR-939	↑	Human	serum	——	Diagnosis of HCC from LC	(222)
		miR-10b-5p, miR-221-3p, miR-223-3p, miR-21-5p	↑	Human	plasma	0.86	Diagnosis of HCC from CH or LC	(234)
	lncRNA	lncRNA-HEIH	↑	Human	serum	——	Diagnosis of HCV-associated HCC from CHC	(236)
		LINC02394	↑	Human	serum	0.719	Diagnosis of HCC from CHB	(237)
		LINC00635	↑	Human	serum	0.750	Diagnosis of HCC from CHB	(237)
		LINC00161	↑	Human	serum	0.794	Prediction of HCC growth and metastasis	(238)
		IncRNA-ATB	↑	Human	serum	——	The prognosis of HCC	(239)
		Lnc85	↑	Human	plasma	0.869	Diagnosis of AFP-negative HCC from healthy controls and LC	(163)
		SENP3-EIF4A1	↑	Human,Mice	plasma	0.8028	The diagnosis of HCC	(160)
	circRNA	circFBLIM1	↑	Human,Mice	serum	——	The therapeutic target of HCC	(149)
		circ-0051443	↑	Human,Mice	plasma	0.8089	The diagnosis and therapeutic target of HCC	(161)
		circRNA-100338	↑	Human,Mice	serum	——	The diagnosis and therapeutic target of HCC	(240)
		circUHRF1	↑	Human,Mice	plasma	——	The therapeutic target of HCC	(169)
		circ-DB	↑	Human,Mice	adipocyte	——	The prognosis of HCC	(202)
	proteins	LAPTM4B-35	↑	Human	serum	——	Prediction of recurrence and diagnosis of HCC	(241)
		SMAD3	↑	Human,Mice	peripheral blood	0.70	The diagnosis of HCC	(242)
		RAB5A	↑	Human	serum	——	The diagnosis and therapeutic target of HCC	(243)
		ENO1	↑	Human,Mice	serum	——	The prognosis of HCC	(244)
	Other combinations	miR-122, miR-148a, AFP	↑	Human	serum	0.931	Diagnosis of HCC from LC	(227)
		SMAD3+ATP	↑	Human,Mice	peripheral blood	0.90	The diagnosis of HCC	(242)
		lncRNA-RP11-513I15.6, miR-1262/RAB11A	↑	Human	serum	——	Diagnosis of E-HCC from CHB	(245)
		miRNA-21, lncRNA-ATB	↑	Human	serum	——	The prognosis of HCC, overall survival	(239)
		ENSG00000258332.1, LINC00635, AFP	↑	Human	serum	0.894	The diagnosis and prognosis of HCC	(237)
		AFP、ENST00000248932.1, ENST00000440688.1, ENST00000457302.2	↑	Human	plasma	0.905 0.879	Predict the probability of HCC in the cancer‐free groups Predict the probability of HCC in the CH groups	(246)
Total EVs		Total EV	↑	Human	serum	0.83	Detection of HCC	(225)
		AFP, GPC3, ALB, APOH, FABP1, FGB, FGG, AHSG, RBP4, TF	↑	Human	plasma	0.93	Diagnosis of E-HCC from LC	(247)
		LINC00853	↑	Human	serum	0.956	Diagnosis of E-HCC from CH、LC	(248)

#### 8.1.2 lncRNAs

In recent years, the potential of EVs-derived lncRNAs in the prognosis of HCC has also attracted growing research interest. lncRNAs alter lncRNA expression can contribute to the cancer phenotype by stimulating cell proliferation, angiogenesis, immune evasion, and inhibition of apoptosis. Among them, linc-VLDLR was identified as a lncRNA enriched in EVs that contributes to the cellular stress response (257). ENSG00000248932.1, ENST00000440688.1 and ENST00000457302.2 were significantly increased in HCC patients, suggesting that lncRNAs may predict tumorigenesis and can be used to dynamically monitor HCC metastases (246). The expression of lncRNA-HEIH was higher in patients with HCV (hepatitis C virus)-associated HCC than that of CHC (chronic hepatitis C) patients (236, 258). Some indicated that sEVs levels of ENSG00000258332.1 and LINC00635 in serum were significantly high and it would be more specific and sensitive when they combined with serum AFP to detect HCC (237). Huang X and Kim S et al. suggested that EVs-derived Lnc85 and LINC00853 showed high positivity in AFP-negative patients with early HCC and were significantly better than AFP, respectively, which is particularly relevant to patients with AFP-negative tumors (163, 248). The potential of EVs containing lncRNAs as biomarkers in the process of HCC diagnosis cannot be ignored, and to find more specific markers for HCC is the next research direction.

#### 8.1.3 CircRNA

There is growing evidence that circRNA in EVs has certain advantages in terms of abundance and stability, indicating that they are promising therapeutic targets for HCC. Similar to miRNA and lncRNA, changes in circRNA expression can also affect the occurrence and progression of HCC (259). In addition, circFBLIM1 was significantly expressed in HCC serum sEVs and promoted HCC progression by affecting the miR-338/LRP6 axis (149). Similarly, Bai N et al. found that circFBLIM1 acts as ceRNA to facilitate HCC by sponging miR-346 (260). In contrast, sEVs-circ-0051443 inhibits HCC progression by regulating miR-331-3p/BAK1 (161). Moreover, Huang XY et al. indicated that HUVECs receiving the circRNA-100,338 could boost the metastatic capacity of HCC cells, which may be related to the regulation of angiogenesis (209). Furthermore, serum EVs-circrna-100, 338 in patients with radical hepatic resection HCC are persistently hyperexpressed, dedicating lung metastases and low survival (240). Ultimately, circMTO1 (261), circSETD3 (262), cSMARCA5 (263), and hsa_circ_0068669 (264) also play key roles in HCC and are potential therapeutic targets, but it remains unclear whether these circRNAs and EVs are related.

### 8.2 EVs-Associated Proteins as Biomarkers of Liver Disease

EVs proteins change with the environment and state of liver cells, it can be used directly or indirectly as a biomarker in different liver diseases (265, 266) to predict the progression of the corresponding liver disease (**Table 4**). CYP450-2E1 (227) and protein tyrosine phosphatase receptor (sPTPRG) isoforms associated with EVs are biomarkers of liver injury, and sPTPRG in plasma reflects the extent of liver injury (274, 278). If CD8, CD14, and connective tissue growth factor (CCN2) are highly expressed in EVs, they can be used to assess the degree of liver fibrosis (272, 273, 279). High expression of Apolipoprotein A-1 by EVs elevates liver-specific proteins such as FGB, causing toxic acute liver injury (269). Studies have shown that EVs containing Carboxylesterase-1 and Carboxylesterase-3 can be evaluated for hepatotoxicity (269, 270). JH H et al. indicated that EVs highly express AnnexinV+EpCAM+ASGPR1+CD133+ taMPs, which can be a novel biomarker for HCC and CCA liquid biopsies (225). If MMP-7 is highly expressed in EVs, it could be a marker for the differential diagnosis of CCA (271). Hepatocytes secrete EVs if ASGPR1+, which can be an alternative non-invasive biomarker of portal hypertension in NASH patients (267).

**Table 4:** EVs-associated proteins as biomarkers of liver disease.

Liver disease	Biomarkers	Types	Function	References
Non-alcoholic steatohepatitis(NASH)	ASGPR1+	Protein	A surrogate noninvasive biomarker of portal hypertension in patients with cirrhotic NASH.	(267)
	CD4+	Protein	Biomarkers of nonalcoholic fatty liver(NAFL)and CHC	(268)
Toxic acute liver injury	Apolipoprotein A-1	Protein	Tentative hepatotoxic markers during hepatic damage	(269)
	Carboxylesterase-1	Protein	Hepatotoxic markers during hepatic damage	(269)
	Carboxylesterase-3	Protein	Non-invasive indicator of drug toxicity	(270)
CCA	AnnexinV+EpCAM+ASGPR1+CD133+ taMPs	–	A novel biomarker of HCC and CCA liquid biopsy	(225)
	MMP-7	Protein	Biomarkers for the diagnosis of CCA	(271)
Liver fibrosis	CD8+	Protein	A biomarker for liver fibrosis	(272)
	CD14+	Protein	A tamps biomarker for liver fibrosis	(273)
Alcoholic steatohepatitis(ASH)	CYP450-2E1	Cytochrome	A potential biomarker for liver injury	(274)
	CD40L	Protein	A potential biomarker for ASH	(275)
Alcoholic hepatitis	CD34+ ASGPR	Protein	Biomarkers of alcoholic hepatitis	(276)
	CK18	Protein	Biomarkers of alcoholic hepatitis	(277)

High CD4+ expression in EVs can be a biomarker to diagnosis nonalcoholic fatty liver (NASH) from chronic hepatitis C (CHC) (268). Positive CD34+ with ASGPR (heavy alcoholic hepatitis) or CK18 (alcoholic hepatitis) in EVs can be used as biomarkers (276, 277), among them, CD34 can also be used as a biomarker to determine heavy alcoholic hepatitis (276). ENO1 upregulates the expression of integrin α6β4 and activates the FAK/Src-p38MAPK pathway (244). Gorji-Bahri G et al. suggested that RAB5A knockdown could be used as a therapeutic target to control the progression of HCC (243). Pang Y et al. saying that LAPTM4B-35 is associated with the HCC relapse, drug resistance, and it is expected to be a new diagnostic marker for HCC (241).

Many studies have shown that EVs affect the progression of various liver diseases by regulating cellular functions and activating key signaling pathways in receptor cells, obviously, which are newly discovered potential biomarkers, to open up new ways to clinically distinguish different kinds of liver disease. Unfortunately, the role of EVs in the diagnosis, prognosis determination and predictive value of liver diseases is still lacking sufficient clinical evidence. Studies on the sensitivity and specificity of these markers in liver disease have also been reported relatively rarely, and relevant applications remain to be further investigated.

## 9 Vesicle-Loaded Drugs

### 9.1 EVs are Natural Nanocarriers

EVs are endogenous cell-derived membranous structures, natural nanocarriers with very low cytotoxicity and immunogenicity, protecting the transported RNA from disassembly and phagocytosis by ribonucleases, with inherent activity targeting and ability to cross biological barriers (30). EVs can transport a wide variety of bioactive molecules, thus altering the physiological functions of the recipient cells and reducing the accumulation of chemotherapeutic drugs in non-target organs, thereby reducing off-target toxicity. Additionally, EVs can bind to each other through various ligand receptors, especially cytokinesis (280). EVs are efficient as synthetic nanocarriers. EVs as nucleic acid and drug delivery vehicles has been extensively studied (281, 282). Notably, EVs as drug carriers need to find an efficient method as cargo loading. Different techniques such as electroporation (283), incubation (284), sonication (285), and freeze-thawing have been applied for the EVs loading (286). What’s more, EVs can also be loaded with specific cargoes with endogenous mechanisms such as direct transfection or co-incubation to deliver the cargo to the cytoplasm (287, 288). However, these loading techniques may lead to some changes in the morphological characteristics and physicochemical properties of EVs, as well as aggregation of themselves or of the cargo they carry (289, 290). A more accurate understanding of the proteomic profile of EVs and the factors influencing protein composition will facilitate the development of protein-based therapeutic strategies for EVs in the future (291, 292).

### 9.2 Application of Drug-Carrying EVs in HCC

We review emerging strategies for targeted delivery using EVs and explore the use of them for the treatment of hepatocellular carcinoma. Treatment of H22 cells with the chemotherapeutic drug methotrexate (MTX) and irradiation with UV light, which could secrete Microparticles (MPs) when co-incubate with the remaining H22 cells, effectively kill tumor cells and reduce adverse effects, while impeding drug efflux (98). We read that RBC-EVs loaded with doxorubicin or sorafenib showed enhanced therapeutic effects in mouse models of *in situ* HCC through a macrophage-dependent mechanism compared with conventional doses of doxorubicin and sorafenib (293). More importantly, drug-loaded RBC-EVs did not show systemic toxicity, whereas conventional doses of doxorubicin and sorafenib did. The main challenges in the current clinical application of EVs are the limited yield and the susceptibility to contamination of EVs with various centrifugation methods (105, 114), which affects the purity and biological properties of EVs. In addition, although EVs are good natural carriers, how to load substances efficiently such as antitumor drugs or genes into EVs is still an urgent technical problem to be solved. Drug-carrying EVs are promising for clinical applications in the treatment of liver diseases, and careful selection of cells of origin for EVs, the creation of appropriate methods for loading the molecules they carry, overcoming low yields, etc. are current research hotspots.

## 10 Discussion

EVs are sensory molecules for information exchange between tumor cells in the microenvironment, activating different signaling pathways and influencing the development, progression and metastasis of tumors (294–297). In recent years, EVs have become promising vehicles in liver disease for their low toxicity, high stability and preferential absorption (298). Today, the application of EVs is still in its early stages. Although there have been clinical trials choosing miRNAs for liver disease, they are still not available for clinical use (298), lacking a number of clinical trials to demonstrate the effectiveness of EVs. The mechanisms and clinical applications of EVs in liver disease need to be studied in more depth. EVs may be an effective intervention in the future, showing a new light for oncology patients. What’s more, EVs can also alter the function of recipient cells and is crucial in the genesis, development and pathogenesis of HCC. Circulating EVs, as a novel signaling modality, which are involved in multiple processes including tumor development and metastatic drug resistance, are promising biomarkers for diagnosing liver disease and monitoring treatment response (46).

Notably, our current understanding of EVs is still inadequate and standard methods for isolating and tracking EVs are lacking. EVs are nearly released by all cells in the body, and many mechanisms involved in their production, transport, uptake and involvement in cancer development have not been fully explored (299), and challenges remain in the extraction, identification and processing of EVs biomarkers for analysis. In addition, the complexity of the immune response and microenvironment in the liver poses a significant challenge to the routine treatment of patients with HCC (300). Therefore, it is important to improve isolation techniques, tracking methods, screening for tissue-specific markers of EVs or the identifying EVs of tissue-specific origin in lesions. Making full use of the different extraction techniques available and optimising them is an important next step in research. In addition, experiments *in vitro* and *in vivo* on EVs still have many limitations, so there is an urgent need to establish well-developed experimental models to further explore their properties and mechanisms of action, and to explore the potential of using this intercellular communication modality in the TME for molecular diagnosis and targeted therapy of tumors. In conclusion, current studies indicate that EVs is crucial in mediating the progression of liver disease and therefore can be thought as a potential therapy for HCC. With a more comprehensive understanding of EVs, more valuable references will be provided for the prevention, diagnosis and prognosis of HCC.

## Author Contributions

JW: conceptualization, methodology, writing-original draft, writing-review & editing, visualization and supervision. XW: writing-original draft, formal analysis and resources. XZ: writing-original draft, formal analysis and project administration. TS: writing-original draft, software. YL: writing-original draft, data curation. WW: writing-original draft, methodology. YH: Conceptualization and Supervision. All authors contributed to the article and approved the submitted version.

## Conflict of Interest

The authors declare that the research was conducted in the absence of any commercial or financial relationships that could be construed as a potential conflict of interest.

## Publisher’s Note

All claims expressed in this article are solely those of the authors and do not necessarily represent those of their affiliated organizations, or those of the publisher, the editors and the reviewers. Any product that may be evaluated in this article, or claim that may be made by its manufacturer, is not guaranteed or endorsed by the publisher.

## Abbreviations

ADC, apparent diffusion coefficient; ADMSC, adipose-derived mesenchymal stem cells; AF4, Asymmetrical flow field-flow fractionation; AHSG, alpha 2-HS glycoprotein; ALB, albumin; ALT, alanine aminotransferase; APOH, apolipoprotein H; ASGPR1, asialoglycoprotein receptor; ASH, Alcoholic steatohepatitis; AST, aspartate aminotransferase; AUROC, Area under Receiver Operating Characteristics; BCA, bicinchonic acid; BM-MSCs, Bone marrow mesenchymal stem cells; CAFs, Cancer-associated fibroblasts; CCA, Cholangiocarcinoma; CCN2, Connective tissue growth factor; ceRNA, competing endogenous RNA; CFH, Complement Factor H; CH, chronic hepatitis; CHB, chronic hepatitis B; CHC, chronic Hepatitis C; CHOP, enhancer-binding protein homologous protein; CK18, Cytokeratin-18; CLEC3B, C-Type Lectin Domain Family 3 Member B; CSCs, Cancer stem cells; CTGF, connective tissue growth factor; DCP, des-gamma-carboxy prothrombin; DDS, drug delivery system; EVs, Extracellular vesicles; EC, endothelial cells; EGF, endothelial growth factor; E-HCC, early-stage hepatocellular carcinoma; EMT, Epithelial–mesenchymal transition; ENO1, Alpha-enolase; EpCAM, epithelial cell adhesion molecule; ESCRT, endosomal sorting complex required for transport; ELISA, enzyme linked immunosorbent assay; FCS, fluorescence correlation spectroscopy; FABP1, fatty acid binding protein 1; FGB, fibrinogen beta chain; FUS, focused ultrasound; GEVs, Glioma-derived EVs; GGT, glutamyl aminotransferase; GPC3, glypican3; HCC, hepatocellular carcinoma HCV, Hepatitis C Virus; HDL, High-density lipoprotein particles; HEMs, Adult human epidermal melanocytes; HGF, Hepatocyte growth factor; HIF-1α, Hypoxia Inducible factor 1 α; HIF-2α, Hypoxia Inducible factor 2 α; HSCs, Hepatic stellate cells; HUVECs, Human umbilical vein endothelial cells; IL, nterleukin; ILVs, intraluminal vesicles; iNOS, Inducible nitric oxide synthase; LAMP2B, lysosomal associated membrane protein 2B; LC, liver cirrhosis; LDLT, living donor liver transplantation; LG3BP, galectin-3-binding protein; LFIA, Lateral-Flow Immunochromatographic Assay; MMP, Matrix metalloproteinase; MPs, Microparticles; MSC, mesenchymal stem cells; MVBs, multivesicular bodies; m/lEVs, medium/large EVs; MDVs, Mitochondria-Derived Vesicles; MAC, membrane attack complex; NTA, nanoparticle tracking analysis; NAFL, Nonalcoholic fatty liver; NASH, non-alcoholic steatohepatitis; NC, normal control; NVs, Nanovesicles; PIGR, polymeric immunoglobulin receptor; RBP4, retinol binding protein 4; ROS, reactive oxygen species; sEVs, small EVs; SNX9, sorting nexin 9; SDS-PAGE, sodium dodecyl sulfate polyacrylamide gel electrophoresis; SAH, Severe alcoholic hepatitis; SMAD3, SMAD Family Member 3; sPTPRG, Protein tyrosine phosphatase receptor Gamma; TAMs, Tumor-associated macrophages; TF, transferrin; TGF-β, Transforming growth factor; TME, Tumor microenvironment; TNFα, Tumor necrosis factor alpha; TSG101, tumor susceptibility gene 101 protein; TRPS, tunable resistive pulse sensing; VEGF, Vascular endothelial growth factor.

## Glossary

**Table:**

ADC	apparent diffusion coefficient
ADMSC	adipose-derived mesenchymal stem cells
AF4	Asymmetrical flow field-flow fractionation
AHSG	alpha 2-HS glycoprotein
ALB	albumin
ALT	alanine aminotransferase
APOH	apolipoprotein H
ASGPR1	asialoglycoprotein receptor
ASH	Alcoholic steatohepatitis
AST	aspartate aminotransferase
AUROC	Area under Receiver Operating Characteristics
BCA	bicinchonic acid
BM-MSCs	Bone marrow mesenchymal stem cells
CAFs	Cancer-associated fibroblasts
CCA	Cholangiocarcinoma
CCN2	Connective tissue growth factor
ceRNA	competing endogenous RNA
CFH	Complement Factor H
CH	chronic hepatitis
CHB	chronic hepatitis B
CHC	chronic Hepatitis C
CHOP	enhancer-binding protein homologous protein
CK18	Cytokeratin-18
CLEC3B	C-Type Lectin Domain Family 3 Member B
CSCs	Cancer stem cells
CTGF	connective tissue growth factor
DCP	des-gamma-carboxy prothrombin
DDS	drug delivery system
EVs	Extracellular vesicles
EC	endothelial cells
EGF	endothelial growth factor
E-HCC	early-stage hepatocellular carcinoma
EMT	Epithelial–mesenchymal transition
ENO1	Alpha-enolase
EpCAM	epithelial cell adhesion molecule
ESCRT	endosomal sorting complex required for transport
ELISA	enzyme linked immunosorbent assay
FCS	fluorescence correlation spectroscopy
FABP1	fatty acid binding protein 1
FGB	fibrinogen beta chain
FUS	focused ultrasound
GEVs	Glioma-derived EVs
GGT	glutamyl aminotransferase
GPC3	glypican3
HCC	hepatocellular carcinoma
HCV	Hepatitis C Virus
HDL	High-density lipoprotein particles
HEMs	Adult human epidermal melanocytes
HGF	Hepatocyte growth factor
HIF-1α	Hypoxia Inducible factor 1 α
HIF-2α	Hypoxia Inducible factor 2 α
HSCs	Hepatic stellate cells
HUVECs	Human umbilical vein endothelial cells
IL	nterleukin
ILVs	intraluminal vesicles
iNOS	Inducible nitric oxide synthase
LAMP2B	lysosomal associated membrane protein 2B
LC	liver cirrhosis
LDLT	living donor liver transplantation
LG3BP	galectin-3-binding protein
LFIA	Lateral-Flow Immunochromatographic Assay
MMP	Matrix metalloproteinase
MPs	Microparticles
MSC	mesenchymal stem cells
MVBs	multivesicular bodies
m/lEVs	medium/large EVs
MDVs	Mitochondria-Derived Vesicles
MAC	membrane attack complex
NTA	nanoparticle tracking analysis
NAFL	Nonalcoholic fatty liver
NASH	non-alcoholic steatohepatitis
NC	normal control
NVs	Nanovesicles
PIGR	polymeric immunoglobulin receptor
RBP4	retinol binding protein 4
ROS	reactive oxygen species
sEVs	small EVs
SNX9	sorting nexin 9
SDS-PAGE	sodium dodecyl sulfate polyacrylamide gel electrophoresis
SAH	Severe alcoholic hepatitis
SMAD3	SMAD Family Member 3
sPTPRG	Protein tyrosine phosphatase receptor Gamma
TAMs	Tumor-associated macrophages
TF	transferrin
TGF-β	Transforming growth factor
TME	Tumor microenvironment
TNFα	Tumor necrosis factor alpha
TSG101	tumor susceptibility gene 101 protein
TRPS	tunable resistive pulse sensing
VEGF	Vascular endothelial growth factor
